# Risk Factors for Surgical Site Infection After Cardiac Surgery in Neonates: A Case–Control Study

**DOI:** 10.3390/jcm13247755

**Published:** 2024-12-19

**Authors:** Vitaliy V. Suvorov, Dmitri O. Ivanov

**Affiliations:** Department of Surgical Diseases of Children, Saint-Petersburg State Pediatric Medical University, 194100 Saint Petersburg, Russia; spb@gpmu.org

**Keywords:** sternal infection, neonate, wound infection in pediatrics, infection in pediatric cardiac surgery, complication in pediatric cardiac surgery, surgical site infections

## Abstract

**Objectives:** This study was undertaken to determine the incidence and risk factors associated with the development of sternal wound infection in neonates after surgery for congenital heart disease. **Methods:** A case–control study was conducted to examine the influence of perioperative risk factors on the development of sternal infection. In total, 253 neonates with CHD underwent a complete median sternotomy. To identify the risk factors influencing the development of sternal infection, multivariable logistic regression analysis was used. **Results:** Overall, 15 (5.9%) patients developed sternal wound infection after surgery. Deep wound infection occurred in six (2.4%) cases. As a result, the perioperative risk factors were the level of oxygen delivery in the postoperative period (OR: 0.956; CI: 0.933–0.98; *p* < 0.001), duration of intubation after surgery (OR: 1.04; CI: 1.003–1.079; *p* = 0.034) and application of surgical manipulation features (OR: 0.0004; CI: 0.000007–0.027; *p* < 0.001). **Conclusions:** The incidence of sternal infection in newborns can be reduced by simple and affordable methods. This will decrease the cost of patient care, length of hospitalization and the risk of secondary complications.

## 1. Introduction

Over the last few decades, pediatric cardiac surgery has undergone significant changes [1]. One of the most significant is the possibility of performing surgery to correct more complex congenital heart disease (CHD) at an early age, often in the neonatal period [2]. Most centers in Russia report good results of neonatal cardiac surgery [3,4,5,6]. However, the development of complications after cardiac surgery, including sternal infection, remain a significant cause of increased duration of mechanical ventilation, treatment in the intensive care unit (ICU) and length of hospitalization, which directly affects mortality [7,8,9,10].

There are few studies that have examined the impact of perioperative risk factors on the development of sternal infection in neonates [11,12,13,14,15]. Some predictors, such as delayed sternal closure or use of peritoneal dialysis, are more common in neonates than in another age group [16]. Therefore, such factors can lead to a longer stay in the intensive care unit in the postoperative period and increase the risk of various complications, including infections [17,18].

Understanding the relationship between outcomes and risk factors is very important because it may influence clinical decision-making by cardiac surgeons, cardiologists, and intensive care physicians when treating this group of patients. Therefore, in this study, we evaluate the influence of perioperative factors on the development of sternal infection in the postoperative period in neonates after surgery for congenital heart disease.

## 2. Materials and Methods

### 2.1. Study Cohort and Study Design

We studied a cohort of pediatric patients with an age < 28 days who underwent surgery at our institution between 1 September 2014 and 30 June 2024. A total of 253 neonates with CHD who underwent cardiac surgery were identified. All neonates underwent a complete median sternotomy. To carry out this study, we analyzed the treatment of patients; we compared those with sternal infection and without it. Within this cohort, a case–control study was conducted to examine the influence of perioperative risk factors on the development of sternal infection. The local ethics committee of St. Petersburg State Pediatric Medical University approved the protocol for this study (No. 29/05; 7 August 2023). Parents of all children were informed about the treatment method, and consent was obtained for the use of clinical data in research studies (examination and treatment).

Inclusion criteria were children with CHD who underwent surgery with complete median sternotomy; age from 0 to 28 days. The criteria for exclusion from the study were other diseases; CHD surgery through other surgical accesses; application of extracorporeal membrane oxygenation; age older than 28 days.

In our study, we evaluated the influence of the following factors on the development of sternal infection in the postoperative period:

− Preoperatively: age, sex, weight, height, genetic abnormalities, diagnosis and cyanotic CHD;
− Intraoperatively: type of surgery, RACHS, repeated sternotomy, intraoperative hypothermia, duration of cardiopulmonary bypass (CPB) and surgery, use of a CPB and features of surgical manipulation.
− Postoperative data in the first 72 h: use of peritoneal dialysis, delayed sternal closure, ICU length of stay, duration of the intubation, use of a CV catheter, hospital stay period, re-sternotomy, daily wound dressings, postoperative bleeding, acute renal failure, daily fluid balance, nutritional status (total protein level, energy needs) and level of oxygen delivery (normal value > 520 mL/min/m^2^).

### 2.2. Features of Surgical Manipulations

**Figure 1:**
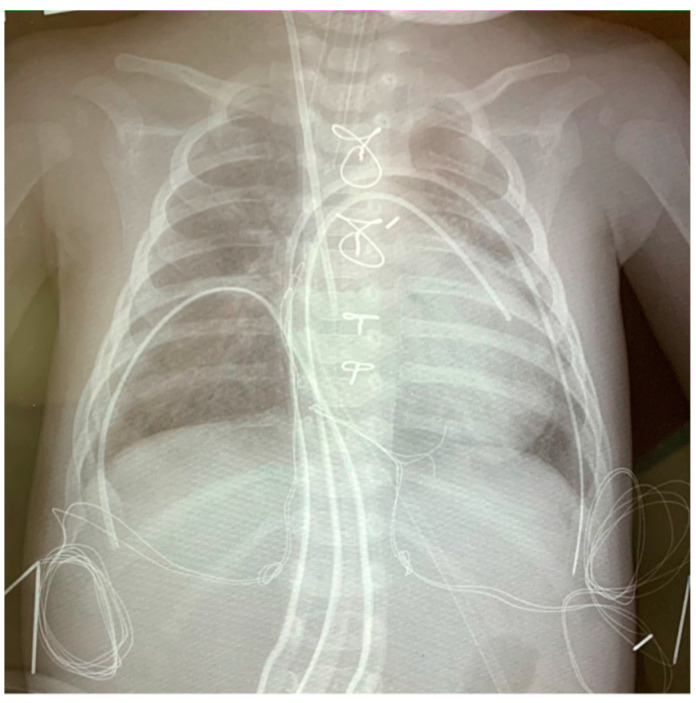
Chest X-ray. The sternum was sutured with a wire ligature, with the formation of an eight-shaped suture on the handle and on the body of the sternum. The pleural cavities and the retrosternal space with pericardium were drained separately.

− A scalpel should be used to dissect soft tissue and periosteum. Electrocoagulation should not be used for this purpose, only when it is necessary to stop bleeding from soft tissues locally.
− It is necessary to avoid impaired blood supply to soft tissues and sternum and careful handling of soft tissues is required [19].
− When performing median sternotomy, dissection of the xiphoid process must be avoided. The use of wax to stop bleeding from the sternum is not recommended. If it is used, it has to be removed before sternal closure.
− Suturing of the sternum should be performed with wire ligature and 1 or 2 eight-shaped sutures (in primary access) should be formed on the manubrium of the sternum (Figure 1). If after repeated sternotomy, it is necessary to make several eight-shaped sutures on the entire sternum [20].
− An antibiotic (vancomycin) in the form of a paste should be applied topically, i.e., 1 g of vancomycin mixed with 2–4 mL of 0.9% NaCl and applied to the sternal incision edges [20,21].
− If there is necrosis of the wound edges, it should be excised from viable tissues before suturing, especially in cases of delayed sternal closure [19,20].
− The fascial-muscular layer should be sutured with separate sutures (use absorbable material).
− It is necessary to irrigate the wound with antibiotic solution during soft tissue closure, i.e., 10 mL of 0.9% NaCl mixed with 0.5–1 g vancomycin [21]. The skin should be sutured with intradermal continuous sutures (preferably with a fast absorbable monofilament suture of the thinnest size and the necessary strength) [19].
− If the wound is covered with a bandage, it is not recommended to change it in the first 24 h and treat it with any solutions (provided that the bandage remains dry). If skin glue is used, no dressings are required for 3–5 days [22].
− When treating the wound, it is necessary to handle it carefully: do not move the edges and press it. It is obligatory to expose the wound to alcohol-containing antiseptic solution for several minutes.

### 2.3. Statistical Analysis

Continuous variables with normal distributions were compared using *t*-tests. For abnormal distributions, differences between the 2 groups were compared using Mann–Whitney U-Test. The chi-square test was applied for compare the qualitative data. To identify the predictors of sternal wound infection after surgery, a logistic regression analysis (univariable and multivariable) was used [23]. The multivariable logistic regressions were conducted using the forward input method (forward LR). The accuracy of prediction of each factor was assessed using receiver-operating characteristic (ROC) curves with calculation of the area under the curve (AUC). The accuracy of prediction was defined as low (AUC: 0.5–0.7), moderate (AUC: 0.7–0.9), and high (AUC: 0.9–1). Statistical processing was carried out using SPSS for Windows (Version 25.0, IBM Corp., Armonk, NY, USA). The level of statistical significance was accepted as *p* < 0.05.

## 3. Results

Overall, 15/253 (5.9%) patients developed sternal wound infection after surgery. Deep wound infection occurred in six (2.4%) cases. A more detailed characterization of the patients is presented in Table 1.

According to univariable regression analysis, the statistically significant predictors in our study were RACHS value (*p* = 0.042), level of an intraoperative hypothermia (*p* = 0.041), duration of surgery (*p* < 0.001), duration of CPB (*p* = 0.004), features of surgical manipulation (<0.001), postoperative use of peritoneal dialysis (*p* = 0.001), delayed sternal closure (*p* = 0.01), acute renal failure after surgery (*p* < 0.001), duration of the intubation (*p* = 0.049), duration of the hospitalisation (*p* = 0.01), duration of use of a CV catheter (*p* = 0.007), postoperative total protein level > 45 g/L (*p* = 0.008), postoperative adequate supply of energy needs (>100 kkal/kg/d) (*p* = 0.002) and level of oxygen delivery (*p* < 0.001). The risk factors in the preoperative period were not statistically significant, so these factors were not included in the multivariable regression analysis.

Multivariable regression analysis revealed that two postoperative and one intraoperative predictors were statistically significant, and determined the probability of sternal infection in newborns after cardiac surgery (Table 2). These factors are as follows: level of oxygen delivery in postoperative period, where each ml/min/m^2^ reduced the risk of sternal infection (OR: 0.956; CI: 0.933–0.98; *p* < 0.001); application of the described features of surgical manipulations, which also decreased the risk of developing a complication (OR: 0.0004; CI: 0.000007–0.027; *p* < 0.001); and duration of the intubation after surgery, where each day increased the risk of sternal infection (OR: 1.04; CI: 1.003–1.079; *p* = 0.034).

**Figure 2:**
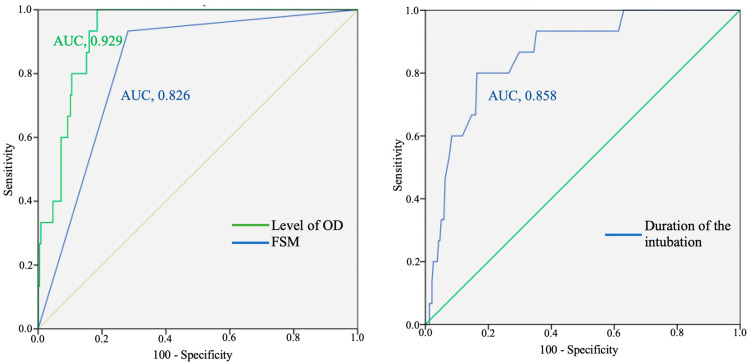
Receiver-operating characteristic (ROC) curves of risk factors of sternal infections with calculation of the area under the curve (AUC). Notes: FSM, Features of surgical manipulation; OD, oxygen delivery.

ROC analysis of the level of oxygen delivery in the postoperative period showed high prognostic values in predicting the risk of developing sternal infection (AUC: 0.929; CI: 0.89–0.967; *p* < 0.001). The accuracy of prediction for two factors were moderate and statistically significant: duration of the intubation in postoperative period (AUC: 0.858; CI: 0.773–0.944; *p* < 0.001) and application of surgical manipulation features (AUC: 0.826; CI: 0.739–0.913; *p* < 0.001) (Figure 2).

## 4. Discussion

According to the data of publications, the development of sternal infection after correction of congenital heart disease using median sternotomy is no more than 15% [6,12,13,14,16,24]. In our study, we focused on the influence of perioperative factors on the development of this complication in newborn children. This is due to the fact that the influence of most factors in the neonatal period differs significantly from the influence of the same factors compared to older children, especially adolescents. Moreover, cases of sternal infection are more common in neonates in most studies [6,13,16,18,25].

Some risk factors are rare in patients older than one month, such as delayed sternal closure and use of peritoneal dialysis. These predictors are often statistically significant in studies. In one study of the causes of sternal infection in neonates undergoing cardiac surgery with delayed sternal closure, the presence of foreign bodies in the wound (e.g., drainage), duration of aortic clamping, use of extracorporeal membrane oxygenation, age and weight at the time of surgery, gestational age, and duration of “open chest” were identified as major factors [16]. We included these predictors in our statistical analysis, but they had no significant effect on the development of this complication in our cohort.

In one article, von Stumm M. et al. analyzed the risk factors for sternal infection in neonates and infants with CHD who underwent surgery [26]. The study included 358 patients, among whom 37% (about 137 patients) were neonates. The incidence of wound infection was 7.3% (26 patients out of 358). However, it is not clear how many neonates developed this complication. It should be noted that delayed sternal closure was reported in 163 cases (45.5%), among which sternal infection occurred in 24 patients. In our study, the patient cohort consists of only neonates because they have significantly more risk factors for wound infection compared to older children. Among the 253 neonates included in the study, 86 (34%) underwent cardiac surgery with delayed sternal closure. Sternal infection developed in 10 patients in this subgroup, representing 4%. This confirms the variability in the impact of perioperative factors on the development of this complication. It should also be noted that in the study of von Stumm M. et al., there were patients who underwent ECMO with delayed sternal closure. This undoubtedly significantly increases the risk of wound infection, which is confirmed in this study; 26.9% of children among all cases of sternal infection underwent ECMO.

One of the main reasons for the development of wound infection is insufficient oxygen supply of tissues. Oxygen supply of tissues depends on the following parameters: oxygen content in blood, oxygen delivery, its consumption and degree of utilization by tissues. It is well known that tissue hypoxia is a risk factor for wound infection and impaired wound reparative processes [25,27,28,29]. Disruption of the normal skin barrier requires wounds to remove foreign bodies and resist infection. Neutrophils provide nonspecific immunity and prevent infection. In the absence of infection, they disappear in about 48 h. Nonspecific phagocytosis and intracellular destruction are the main pathways activated in wounds. Non-specific neutrophil immunity depends on high partial pressure of oxygen since reactive oxygen species are the main component of bactericidal defense against wound pathogens [19].

Some studies confirm the fact of a strong effect of hematocrit reduction on the development of hypoxemia, especially as a result of intraoperative and postoperative hemodilution. This is reflected in a lower blood oxygen saturation, which negatively affects not only wound healing, but also renal and cerebral oxygenation [30,31,32].

One of the most interesting systematic reviews on reduction the incidence of infectious complications is the meta-analysis conducted by L. Dalfino et al. in 2011. According to their data, standard approaches to hemodynamic correction in surgical patients may be the cause of infectious complications. In 26 randomized controlled clinical trials including 4188 patients, treatment according to the principles of individualized targeted perioperative hemodynamic correction significantly reduced the incidence of infections, including surgical wound infection [33]. In accordance with these findings, we follow this strategy in children in the perioperative period: we control the volume of fluid administration, daily fluid balance, hematocrit and hemoglobin values (correct them if necessary), evaluate cardiac function using echocardiography and avoid the development of systemic perfusion disorders. Among these general concepts, we pay special attention to ensuring oxygen delivery to tissues. Following to the results of our analysis, the level of oxygen delivery (one of the indicators of this approach) in neonates is highly significant; it essentially reduced the occurrence of sternal infection in the postoperative period (OR: 0.001; CI: 0.0003–0.067; *p* = 0.002).

Another multicenter study, which complements the previous one, analyzed the effect of daily fluid balance on the development of complications in acute kidney injury [34]. Obviously, fluid balance was higher in patients with acute kidney injury, which not only led to the development of complications and aggravated renal failure, but also was associated with 28-day mortality. Moreover, according to our data, acute kidney injury significantly increased the risk of the studied complication—sternal infection. It should be noted that the features of surgical interventions we mention are directly related to these factors. They allow enhanced wound healing by minimizing the risk of impaired tissue perfusion in the wound area. This may be associated with the progression of tissue edema, impaired delivery of oxygen and nutrients to the tissues, altered immune response in this compromised area, decreased cardiac pumping function, etc. In our study, this result was also supported by the statistically significant effect of a surgical component: application of surgical manipulation features (OR: 0.015; CI: 0.002–0.108; *p* < 0.001).

### Limitations

This study was conducted at a single center with relatively homogeneous treatments, so the results of our study may have some limitations in the representativeness of the data. For this purpose, a multicenter study should be conducted. Also, in the future, randomization of patients can be performed to more correctly evaluate the influence of various factors on the development of sternal infection in neonates after correction of CHD.

## 5. Conclusions

To minimize the risk of sternal infection in newborns after cardiac surgery, it is advisable to focus on normalization of tissue perfusion and oxygen delivery to tissues and to reduce the duration of ventilation. Therefore, at the intraoperative stage, surgical measures make a great contribution, and in the postoperative period, the main attention should be on an individualized approach to intensive care. Thus, it is possible to prevent the development of sternal infection in newborns by using simple, inexpensive and readily available tools and methods.

## Figures and Tables

**Table 1:** Demographic and clinical characteristics of the patients with analysis of factors associated with development of sternal infection (univariable regression analysis).

Characteristics		Sternal Infection		*p*-Value
	All (n = 253/%)	Yes (n = 15/%)	No (n = 238/%)	
Preoperative clinical characteristics				
Height, sm (Md)	51 [49; 52]	51 [49; 52]	51 [49; 53]	0.411
Weight, kg (Md)	3.3 [2.9; 3.7]	3.3 [2.9; 3.7]	3.7 [3; 3.9]	0.088
Sex (male)	150/59,3	10/3,95	140/55,3	0.976
Age at the time of surgery, days (Md)	10 [6; 19]	10.5 [6; 19]	7 [4; 14]	0.133
Type of CHD:				0.381
CoA + Arch hypoplasia/IAA/VR	54/21.3	1/0.4	53/20.9	
D-TGA/D-TGA + VSD	40/15.8	4/1.6	36/14.2	
VSD/AVCD/TF	19/7.5	0/0	19/7.5	
Pulmonary atresia/truncus arteriosus	16/6.3	0/0	16/6.3	
HLHS/single ventricle	80/31.6	8/3.2	72/28.5	
DORV	14/5.5	1.0.4	13/5.1	
TAPVR	12/4.7	0/0	12/4.7	
AV or MV stenosis/ALCAPA	13/5.1	1/0.4	12/4.7	
other	5/2	0/0	5/2	
Cianotic CHD, overall	89/35.2	7/2.8	82/32.4	0.341
Genetic abnormalities, overall	26/10.3	2/0.8	24/9.5	0.689
Intraoperative clinical characteristics				
Type of operation:				0.778
Aortic arch reconstruction	32/12.6	0/0	32/12.6	
Aortic arch reconstruction + other	28/11.1	3/1.2	25/9.9	
Arterial switch	22/8.7	3/1.2	19/7.5	
Arterial switch + VSD closure/Rastelli	18/7.1	1/0.4	17/6.7	
ASD closure/atrioseptostomy	0/0	0/0	0/0	
Valve plasty/replacement	9/3.6	0/0	9/3.6	
Valve plasty/replacement + other	3/1.2	0/0	3/1.2	
VSD or AVCD repair or pacemaker implantation	7/2.8	0/0	7/2.8	
RV-PA conduit impl./VR repair	7/2.8	1/0.4	6/2.4	
Bilateral PA banding/PA banding	73/28.9	3/1.2	70/27.7	
Norwood/DKS	19/7.5	4/1.6	15/5.9	
Other correction of a single ventricle	19/7.5	0/0	19/7.5	
TA/ALCAPA repair/PA unifocalisation	4/1.6	0/0	4/1.6	
PV plasty/PV reimplantation	12/4.7	0/0	12/4.7	
RACHS:				0.042
1	0/0	0/0	0/0	
2	5/2	0/0	5/2	
3	128/50.6	7/2.8	121/47.8	
4	100/39.5	4/1.6	96/37.9	
5	0/0	0/0	0/0	
6	20/7.9	4/1.6	16/6.3	
Repeat_sternotomy, overall	6/2.4	0/0	6/2.4	0.511
Hypothermia intraoperatively:				0.041
14–20 degrees Celsius	19/7.5	4/1.6	15/5.9	
20–28 degrees Celsius	56/22.1	4/1.6	52/20.6	
28–34 degrees Celsius	42/16.6	3/1.2	39/15.4	
Normal degrees Celsius	136/53.8	4/1.6	132/52.2	
Duration of surgery, min (Md)	290 [180; 400]	290 [180; 391.2]	540 [300; 600]	<0.001
Cardio-pulmonary bypass, duration, min (Md)	110 [0; 190]	100 [0; 185]	240 [110; 305]	0.004
Cardio-pulmonary bypass, overall	170/67.2	158/62.2	12/4.7	0.285
Features of surgical manipulation, overall	173/68.4	2/0.8	171/67.6	<0.001
Postoperative clinical characteristics				
Peritoneal dialysis, overall	87/34.4	12/4.7	75/29.6	0.001
Resternotomy after surgery, overall	9/3.6	1/0.4	8/3.2	0.511
Delay sternal closure, overall	86/34	10/4	76/30	0.01
Bleeding after surgery, overall	10/4	1/0.4	9/3.6	0.538
Acute renal failure after surgery, overall	55/21.7	9/3.6	46/18.2	<0.001
Intensive care unit stay, days (Md)	16 [9; 25]	14.5 [9; 23]	33 [26; 42]	0.174
Duration of the intubation, days (Md)	10 [6; 18]	10 [5; 18]	27 [20; 37]	0.049
Duration of the hospitalisation, days (Md)	25 [17; 37]	24 [15; 35]	47 [40; 53]	0.01
Duration of use of a central venous catheter, days (Md)	20 [12; 34]	19 [12; 31]	43 [38; 54]	0.007
Daily wound dressings, overall	174/68.8	15/5.9	159/62.8	0.97
Positive daily fluid balance (>50 mL/kg/d), overall	102/40.3	15/5.9	87/34.4	0.957
Total protein level (>45 g/L), overall	182/71.9	6/2.4	176/69.6	0.008
Energy needs (>100 kkal/kg/d), overall	173/68.4	4/1.6	169/66.8	0.002
Level of oxygen delivery, ml/min/m^2^	552 [505; 595]	554 [516.5; 597]	432 [354; 474]	<0.001

Notes: CHD, congenital heart disease; VSD, ventricular septal defect; AVSD, atrioventricular septal defect; TAPVR, total anomalous pulmonary venous return; D-TGA, D-transposition of the great arteries; AV, aortic valve; ALCAPA, anomalous left coronary artery from the pulmonary artery; TF, tetralogy of Fallot; DORV, double-outlet right ventricle; HLHS, hypoplastic left heart syndrome; CoA, coarctation of the aorta; TA, truncus arteriosus; IAA, interrupted aortic arch; VR, vascular ring; DKS, Damus–Kaye–Stansel procedure; RV, right ventricle; PA, pulmonary artery; RACHS, risk adjustment for congenital heart surgery.

**Table 2:** Results of the initial assessment of the impact of risk factors of sternal infections (multivariable regression analysis).

Risk Factors	Multivariable Regression Analysis	
	OR [CI 95%]	*p*-Value *
Features of surgical manipulation	0.0004 [0.00007–0.027]	<0.001
Level of oxygen delivery in PO	0.956 [0.933–0.98]	<0.001
Duration of the intubation, PO	1.04 [1.003–1.079]	0.034

Notes: * signifies a statistically significant value (*p* < 0.05). OR, odds ratio; CI, confidence interval; PO, postoperative.

## Data Availability

The datasets generated during and/or analyzed during the current study are available from the corresponding author upon reasonable request.
